# DFT and TD-DFT calculation of new thienopyrazine-based small molecules for organic solar cells

**DOI:** 10.1186/s13065-016-0216-6

**Published:** 2016-10-27

**Authors:** Mohamed Bourass, Adil Touimi Benjelloun, Mohammed Benzakour, Mohammed Mcharfi, Mohammed Hamidi, Si Mohamed Bouzzine, Mohammed Bouachrine

**Affiliations:** 1 ECIM/LIMME, Faculty of Sciences Dhar El Mahraz, University Sidi Mohamed Ben Abdallah, Fez, Morocco; 2 Equipe d’Electrochimie et Environnement, Faculté des Sciences et Techniques, University Moulay Ismaïl, Meknes, Morocco; 3 Centre Régional des Métiers d’Education et de Formation, BP 8, Errachidia, Morocco; 4 ESTM, (LASMAR), University Moulay Ismaïl, Meknes, Morocco

**Keywords:** π-conjugated molecules, Thienopyrazine derivatives, Organic solar cells, TD-DFT, Optoelectronic properties, Voc (open circuit voltage)

## Abstract

**Background:**

Novel six organic donor-π-acceptor molecules (D-π-A) used for Bulk Heterojunction organic solar cells (BHJ), based on thienopyrazine were studied by density functional theory (DFT) and time-dependent DFT (TD-DFT) approaches, to shed light on how the π-conjugation order influence the performance of the solar cells. The electron acceptor group was 2-cyanoacrylic for all compounds, whereas the electron donor unit was varied and the influence was investigated.

**Methods:**

The TD-DFT method, combined with a hybrid exchange-correlation functional using the Coulomb-attenuating method (CAM-B3LYP) in conjunction with a polarizable continuum model of salvation (PCM) together with a 6-31G(d,p) basis set, was used to predict the excitation energies, the absorption and the emission spectra of all molecules.

**Results:**

The trend of the calculated HOMO–LUMO gaps nicely compares with the spectral data. In addition, the estimated values of the open-circuit photovoltage (V_oc_) for these compounds were presented in two cases/PC_60_BM and/PC_71_BM.

**Conclusion:**

The study of structural, electronics and optical properties for these compounds could help to design more efficient functional photovoltaic organic materials.

## Background

The organic bulk heterojunction solar cells (BHJ) are considered as one of the promising alternative used for renewable energy. This is attributed to their several advantages to fabricate the flexible large-area devices and also to their low cost compared to other alternatives based on inorganic materials [1, 2]. Generally, the organic BHJ solar cells based on the mixture of electron donor (material organic) and electron acceptor materials as PCBM or its derivatives and have been utilized in the aim to harvest the sunlight. Over the past few years, considerable effort has been focused on improving organic solar cells (OSC) performance to achieve power conversion efficiencies (PCE) of 10%. The following strategies have been adopted for this purpose [3–13]: (1) design of the new photoactive materials able to increase the efficiency of photoconversion such as fullerenes and π-conjugated semiconducting polymers; (2) use of functional layers of buffering, charge transport, optical spacing, etc., and; (3) morphological tuning of photoactive films by post-annealing, solvent drying, or processing by using additives. After many efforts, the design of the organic BHJ solar cells based on polymer semiconducting (PSCs) as an electron donating and PCBM as an electron accepting showed impressive performances in converting solar energy to electrical energy. Finally, the power conversion efficiency (PCE) was improved in the range of 7–9.2% [14–21] for single layer PSCs and 10.6% [14] for tandem structured PSCs. These kinds of solar cells based on polymers have potential applications in next-generation solar cells compared to dye-sensitized solar cells (DSSC) and inorganic thin-film. On the other hand, considerable research has been directed to developing an efficient small-molecule organic used as a semiconductors and to improve their performance in the organic solar cells (OSCs), with the near-term goal of achieving a PCE comparable to that of polymer solar cells (PSCs) [22–24].

Small-molecule organic semi-conductors are more suitable than polymer-based ones for mass production because the latter suffer from poor reproducibility of the average molecular weight, high dispersity, and difficulties in purification. Recently, the small molecule for organic solar cells (SMOSCs) with PCEs exceeding 6% have been reported [25] thus making solution-processed SMOSCs strong competitors to PSCs. This inspires us to develop a new low band gap for small molecules for organic solar cells application. In order to achieve high current density in SMOSCs, utilizing new donor molecules that can efficiently absorb the sunlight at the maximum solar flux region (500–900 nm) of the solar spectrum, because the energy conversion efficiency of the small molecule for organic solar cells is directly attached to the light harvesting ability of the electron donor molecules. In addition, to get high open circuit voltage (Voc), the HOMO levels of the donor molecules should be down a −5.0 eV, in which this factor is calculated by the difference between the HOMO and LUMO levels of the donor and acceptor materials, respectively. The most small molecule organic semiconductors used in solar cells have a push–pull structure comprising electron donors and acceptors in objective to enhance the intramolecular charge transfer (ICT) and the band gap becomes narrow and then, yielding higher molar absorptivity [22–25]. A common strategy to enhance the power conversion efficiency of low band gap conjugated molecules as an alternating (D-A) or (D-π-A) structures because this improves the excitation charge transfer and transport [26]. Different authors described in recent studies the importance of compounds with D-π-A structure and their role in the elaboration of the organic solar cell [27–29]. The organic material based on thienopyrazine has been used as a donor unit; still receive considerable attention for their exceptional optoelectronic properties [30, 31]. Knowledge about the optoelectronic properties of these new materials can help with the design of new materials with optimized properties for solar energy conversion. In our previous works [32, 33], we have reported a theoretical study of photovoltaic properties on a series of D-π-A structures of thienopyrazine derivatives as photoactive components of organic BHJ solar cells.

In order to obtain materials with more predominant capability, the development of novel structures is now being undertaken following the molecular engineering guidelines, the theoretical studies on the electronic structures of these materials have been done in order to rationalization the properties of known ones and the prediction those of unknown ones [26]. As is known, the knowledge of the HOMO and LUMO levels of the materials is crucial in studying organic solar cells. The HOMO and LUMO energy levels of the donor and of the acceptor compounds present an important factor for photovoltaic devices which determine if the charge transfer will be happen between donor and acceptor. The thienopyrazine derivatives would be much more promising for developing the panchromatic materials for photovoltaic, and thus, provide much higher efficiencies if new absorption bands could be created in the visible light region.

**Fig. 1:**
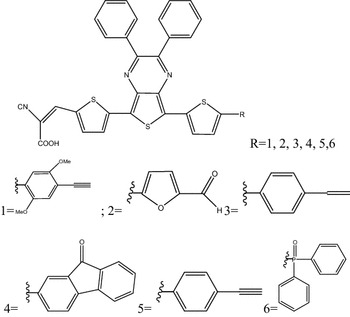
Chemical structure of study compounds Pi (i = 1–6)

In this paper, we report a strategy to control the band-gap and different optoelectronics properties by using the DFT method on a series of no symmetrical branched molecules based on thienopyrazine as a central core and cyanoacrylic acid as the end group connected with different π-conjugated groups Xi, as shown in Fig. 1. We think that the presented study for these compounds listed in Fig. 1 bout their structural, electronic and optical properties could help to design more efficient functional photovoltaic organic materials, for aim to find the best material which is used as a donor electron in BHJ device in the solar cell.

## Computational methods

All calculations were carried out using density functional theory (DFT) with B3LYP (Becke three-parameter Lee–Yang–Parr) exchange-correlation functional [34]. 6-31G(d,p) was used as a basis set for all atoms (C, N, H, O, S). Recently, Tretiak and Magyar [35] have demonstrated that the charge transfer states can be achieved in D-π-A structure a large fraction of HF exchange is used. A newly designed, functional, the long range Coulomb-attenuating method (CAM-B3LYP) considered long-range interactions by comprising 81% of B88 and 19% of HF exchange at short-range and 35% of B88 and 65% of HF exchange at long-range [36]. Furthermore, The CAM-B3LYP has been used especially in recent work and was demonstrated its ability to predict the excitation energies and the absorption spectra of the D-π-A molecules [37–40]. Therefore, in this work, TD-CAM-B3LYP method has been used to simulate the vertical excitation energy and electronic absorption spectra. It is important to take into account the solvent effect on theoretical calculations when seeking to reproduce or predict the experimental spectra with a reasonable accuracy. Polarizable continuum model (PCM) [41] has emerged in the last two decades as the most effective tools to treat bulk solvent effects for both the ground and excited states. In this work, the integral equation formalism polarizable continuum model (IEF-PCM) [42, 43] was used to calculate the excitation energy. The oscillator strengths and excited state energies were investigated using TD-DFT calculations on the fully DFT optimized geometries.

By using HOMO and LUMO energy values for a molecule, chemical potential, electronegativity and chemical hardness can be calculated as follows [44]:

$$\mu = \left( {E_{HOMO} + E_{LUMO} } \right)\;/\; 2$$

Chemical potential

$$\eta = \left( {E_{LUMO} - E_{HOMO} } \right)\;/\; 2$$

(Chemical hardness),

$$\chi =-\;(E_{HOMO} + E_{LUMO} )\;/\;2$$

(electronegativity),

all calculations were performed using the Gaussian 09 package [45].

## Results and discussion

### Ground state geometry

**Fig. 2:**
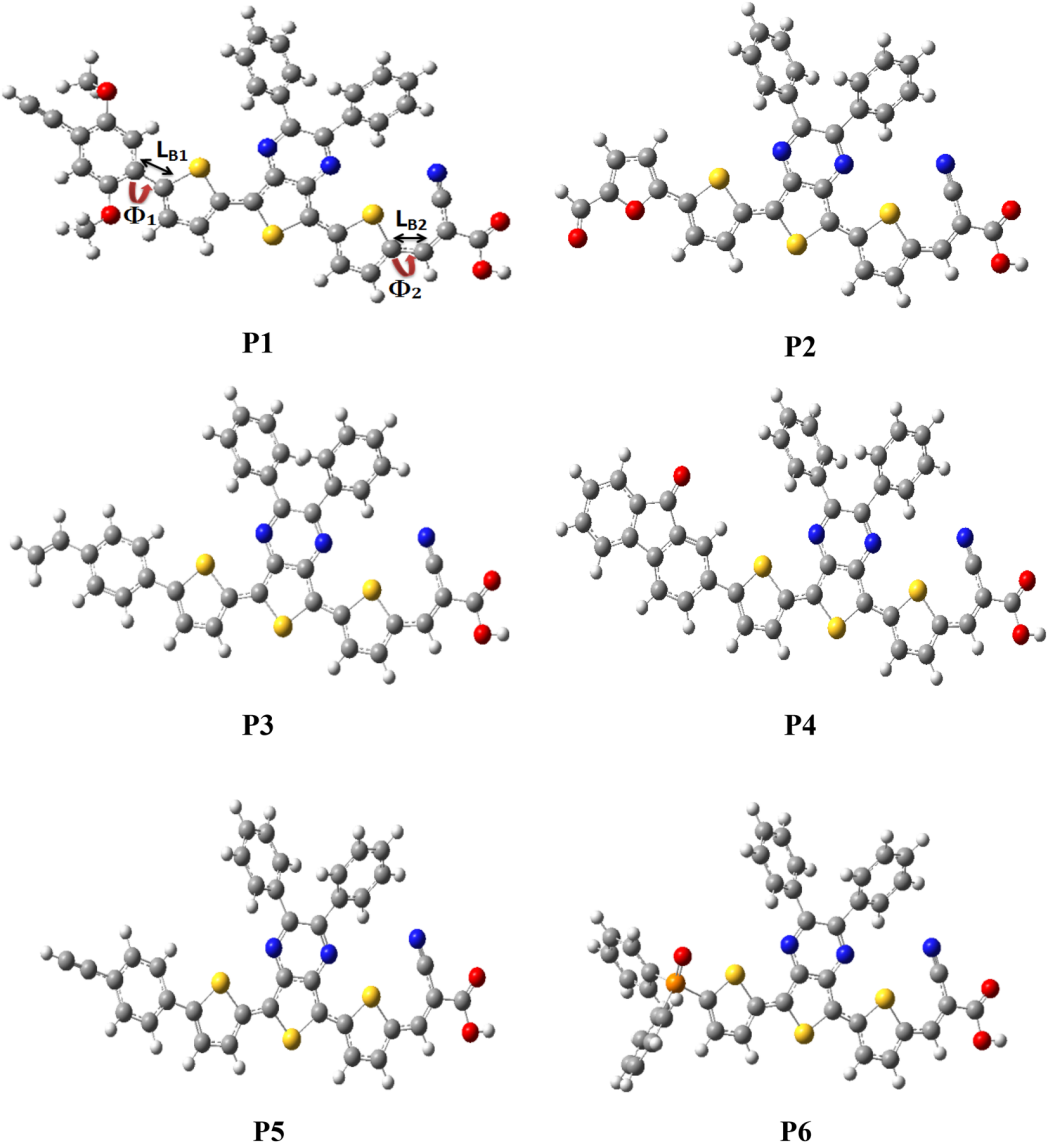
Optimized geometries obtained by B3LYP/6-31G(d,p) of the studied molecules

The optimized structures of all molecules obtained with the B3LYP/6-31G(d,p) level, are presented in Fig. 2.

Figure 2 shows the definition of torsional angles Φ_1_ and Φ_2_ between D and π-spacer A and π-spacer respectively, intramolecular charge transfer (ICT) which is represented by the π-spacer and the bridge bonds between D and π-spacer and A and π-spacer were marked as L_B1_ and L_B2_ respectively, using compound [P1] as an example (see Fig. 2). Torsional angles Φ_1_ and Φ_2_ are the deviation from coplanarity of π-spacer with the donor and acceptor and the L_B1_ and L_B2_ are the bond lengths of π-spacer from the donor and acceptor. The torsional angles (Φ_1_ and Φ_2_), and bridge lengths (L_B1_ and L_B2_) are listed in Table 1.

**Table 1:** Optimized selected bond lengths and bond angles of the studied molecules obtained by B3LYP/6-31G(d,p) level [the unit of bond lengths is angstroms (Å), the bond angles and dihedral angles is degree (°)]

Compounds	S_0_				S_1_			
	L_B1_	L_B2_	Φ_1_	Φ_2_	L_B1_	L_B2_	Φ_1_	Φ_2_
P1	1.463	1.421	19.72	2.77	1.449	1.411	14.17	3.41
P2	1.435	1.423	0.78	2.95	1.425	1.413	0.56	3.98
P3	1.462	1.421	22.19	2.85	1.449	1.411	10.07	3.67
P4	1.463	1.422	22.04	2.82	1.451	1.411	11.61	3.34
P5	1.462	1.422	22.71	2.84	1.452	1.412	12.68	3.53
P6	1.818	1.422	41.37	2.76	1.810	1.412	42.23	3.50

As shown in Table 1, all calculations have been done by using DFT/B3LYP/6-31G(d,p) level. The large torsional angle Φ_1_ of the compounds P1, P2, P3, P4, P5 and P6 suggest that strong steric hindrance exists between the donor and π-spacer.

For P2, the dihedral angles Φ_1_ formed between the donor group and π-spacer is 0.78°, indicating a smaller conjugation effect compared to the other compounds where the coplanarity can be observed, but this geometry of P2 allows inhibiting the formation of π-stacked aggregation efficiently. Furthermore, the dihedral angles Φ_2_ of all compounds is very small (2.77, 2.95, 2.85, 2.82, 2.84 and 2.76) wich indicates that the acceptor (cyanoacrylic unit) is coplanar with π-spacer (thiophene–thienopyrazine–thiophene). In the excited state (S_1_), we remark that the dihedral angles Φ1 for all compounds are significantly decreased in comparison with those in the ground state (S0), except P2 and P6, Φ_1_ is almost similar to that of the ground state. It indicates that the nature of the S1 state of the molecular skeleton of all compounds is different from the S0 state, and the complete coplanarity in S1 state triggers the fast transfer of the photo-induced electron from S0 to S1.

The shorter value from the length of bridge bonds between π-spacer and the donor (L_B1_) and in another side between π-spacer and acceptor (L_B2_) favored the ICT within the D-π-A molecules. However, in the ground state (S_0_) the calculated critical bond lengths L_B1_ and L_B2_ are in the range of 1.421–1.462 Å showing especially more C=C character, except the compound P6, which enhances the π-electron delocalization and thus decreases the L_B_ of the studied compounds and then favors intramolecular charge transfer ICT. On the other hand, upon photoexcitation to the excited state (S_1_), the bond lengths and torsional angles for these compounds significantly decreased in comparison with those in the ground state (S_0_), especially the linkage between the π-spacer and the acceptor moiety (L_B2_). These results indicate that the connection of acceptor group (2-cyanoacrylic acid) and the π-bridge is crucial for highly enhanced ICT character, which is important for the absorption spectra red-shift.

### Electronic properties

Among electronic applications of these materials is their use as organic solar cells, we note that theoretical knowledge of the HOMO and LUMO energy levels of the components is crucial in studying organic solar cells. The HOMO and LUMO energy levels of the donor and of the acceptor components for photovoltaic devices are very important factors to determine whether the effective charge transfer will happen between donor and acceptor. The experiment showed that the HOMO and LUMO energies were obtained from an empirical formula based on the onset of the oxidation and reduction peaks measured by cyclic voltammetry. But in the theory, the HOMO and LUMO energies can be calculated by DFT calculation. However, it is noticeable that solid-state packing effects are not included in the DFT calculations, which tend to affect the HOMO and LUMO energy levels in a thin film compared to an isolated molecule as considered in the calculations. Even if these calculated energy levels are not accurate, it is possible to use them to get information by comparing similar oligomers or polymers.

The calculated frontier orbitals HOMO, LUMO and band gaps by using B3LYP/6-31G(d,p) level of six compounds (P1, P2, P3, P4, P5and P6) are listed in Table 2. The values of HOMO/LUMO energies are −5.025/−3.057 eV for P1, −5.276/−3.293 eV for P2, −5.091/−3.099 eV for P3, −5.139/−3.124 eV for P4, −5.155/−3.140 eV for P5 and −3.140/−3.159 for P6 and corresponding values of energy gaps are 1.968 eV for P1, 1.983 eV for P2, 1.992 eV for P3, 2.015 eV for P4, 2.015 eV for P5 and 2.171 eV for P6. The calculated band gap Eg of the studied model compounds increases in the following order P1 < P2 < P3 < P4 = P5 < P6. The much lower Eg of P1, P2 and P3 compared to that of P6 indicates a significant effect of intramolecular charge transfer, which would make the absorption spectra red shifted. However, the Eg values of P1, P2 and P3 are smaller than that of P6. This is clearly due to the effect of the electron-donor unit which is strong of P1, P2, and P3 than that of other compounds. All molecules present low energy gap are expected to have the most outstanding photophysical properties especially P1.

**Table 2:** Calculated E_HOMO_, E_LUMO_ levels, energy gap (E_g_), dipole moment (ρ) and other quantum parameters chemical as electronegativity (χ), chemical potential (μ) and chemical hardness (η) values of the studied compounds obtained by B3LYP/6-31G(d,p) level

Compounds	E_HOMO_ (eV)	E_LUMO_ (eV)	Eg (eV)	μ (eV)	η (eV)	χ (eV)	ρ (Debye)
P1	−5.025	−3.057	1.968	−4.092	1.866	4.092	8.966
P2	−5.276	−3.293	1.983	−4.2175	2.117	4.218	1.851
P3	−5.091	−3.099	1.992	−4.125	1.932	4.125	6.803
P4	−5.139	−3.124	2.015	−4.149	1.98	4.149	8.980
P5	−5.155	−3.140	2.015	−4.157	1.996	4.157	5.975
P6	−5.33	−3.159	2.171	−4.2445	2.171	4.245	7.552​
PCBM	−6.100	−3.750	*****	−4.925	2.350	4.925	******

### Quantum chemical parameters

Generally, the molecules having a large dipole moment, possesses a strong asymmetry in the distribution of electronic charge, therefore can be more reactive and be sensitive to change its electronic structure and its electronic properties under an external electric field. Through the Table 2, we can observe that the dipole moment (ρ) of compounds P1 and P4 are greater than others compounds, therefore we can say that these compound are more reactive that other compound, indeed, these compounds are more favorite to liberate the electrons to PCBM.

On another side, we note that the PCBM has the smallest value of the chemical potential (μ = −4.9) compared to six compounds (P1, P2, P3, P4, P5, and P6) (see Table 2), this is a tendency to view the electrons to escape from compound Pi has a high chemical potential to PCBM which has a small chemical potential, therefore PCBM behaves as an acceptor of electrons and others compounds Pi behave as a donor of electrons. For the electronegativity, we remark that the PCBM has a high value of electronegativity than other compounds (P1, P2, P3, P4, P5, and P6) (Table 2), thus the PCBM is the compound that is able to attract to him the electrons from others compounds. In another hand, we remark that the PCBM compound has a high value of chemical hardness (η) in comparison with other six compounds, this indicates that the PCBM is very difficult to liberate the electrons, while the other compounds are good candidates to give electrons to the PCBM (see Table 2).

**Fig. 3:**
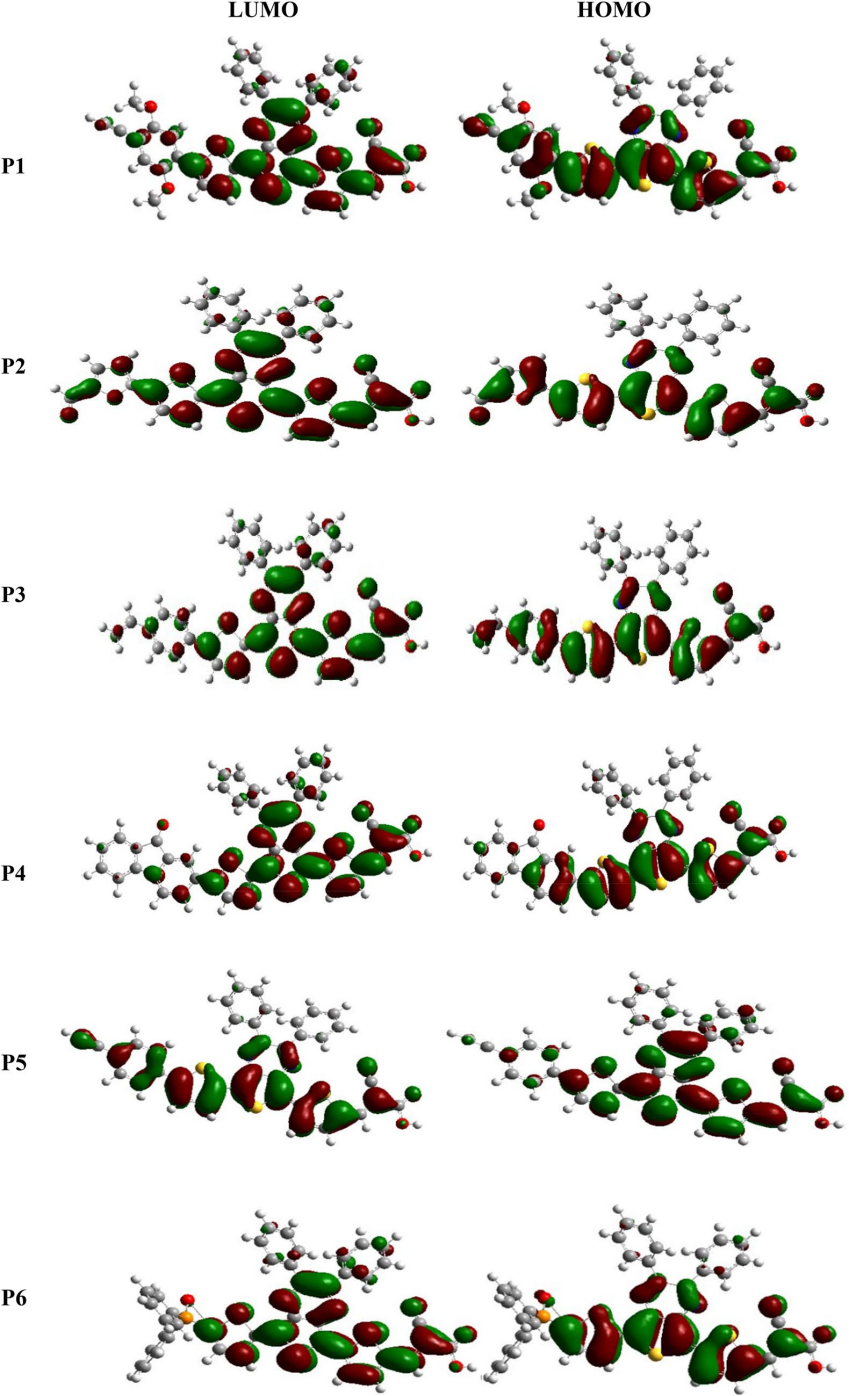
The contour plots of HOMO and LUMO orbitals of the studied compounds Pi

Figure 3 shows the frontier molecular orbitals for all the Six compounds (computed at B3LYP/6-31G(d,p) level). The FMOs of all six models have analogous distribution characteristics. All HOMOs show the typical aromatic features with electron delocalization for the whole conjugated molecule and are mainly localized at the donor parts and conjugated spacer, whereas the LUMOs are concentrated on the π-spacer and at the acceptor moieties (cyano acrylic unit). In another hand, the HOMO possesses an anti-bonding character between the consecutive subunits, while the LUMO of all oligomers shows a bonding character between the two adjacent fragments, so the lowest lying singlet states are corresponding to the electronic transition of π–π* type. Therefore the photoexcited electron will be transferred from donor moiety (donor of an electron) to the acceptor group during the excitation process, which is of benefit to the injection of the photoexcited electrons to the LUMO of the semiconductor (PCBM). In another side, we remark that the acceptor group (–CCNCOOH) of all compound has a considerable contribution to the LUMOs which could lead to a strong electronic coupling with PCBM surface upon photoexcitation electron and thus improve the electron injection efficiency, and subsequently enhance the short-circuit current density J_sc_.

### Photovoltaic properties

Generally, the power conversion efficiency (PCE) is the most commonly used parameter to compare the performance of various solar cells, and to describe it for any compounds, some important parameters has been evaluated such as the short-circuit current density (J_SC_), the open circuit voltage (V_OC_), the fill factor (FF), and the incident photon to current efficiency (P_inc_). The power conversion efficiency (PCE) was calculated according to the following Eq. (1):

1 $$PCE\, = \;\frac{{J_{SC} \;V_{OC} FF}}{{P_{inc} }}$$

where the J_SC_ is estimated by the maximum current which flows in the device under illumination when no voltage is applied, in which dependent on the morphology of the device and on the lifetime and the mobility of the charge carriers [46].

The maximum open-circuit voltage (Voc) of the BHJ is determined by the difference between the HOMO of the donor (π-conjugated molecule) and the LUMO of the acceptor, taking into account the energy lost during the photo-charge generation [47, 48]. It has been found that the V_OC_ is not very dependent on the work functions of the electrodes [49, 50].

The theoretical values of open-circuit voltage Voc of the BHJ solar cell have been calculated from the following expression [47, 48]:

2 $$V_{OC} \; = \;\left| {E_{HOMO}^{Donor} } \right|\; - \;\left| {E_{LUMO}^{Acceptor} } \right|\; - \;0.3$$

where the represents the elementary charge, and the value of 0.3 V is an empirical factor. Scharber et al. [48] proposed the Eq  (2) using −4.3 eV as LUMO energy for the PC_71_BM.

In addition, low LUMO of the π-conjugated compounds and a high LUMO of the acceptor of the electron (PC_71_BM, PC_60_BM) increase the value of V_OC_, which contributes a high efficiency of the solar cells [48, 50].

The theoretical values of the open circuit voltage V_oc_ of the studied molecules range from 1.499 to 1.804 eV in the case of PC_60_BM and 0.425 to 0.73 eV in the case of PC_71_BM (Table 3), these values are sufficient for a possible efficient electron injection into LUMO of the acceptor.

**Table 3:** Energy values of E_LUMO_ (eV), E_HOMO_ (eV), Egap (eV) and the open circuit Voltage V_oc_ (eV) and LUMO_donor_−LUMO_acceptor_of the studied molecules obtained by B3LYP/6-31G(d,p) level

Compounds	E_LUMO_ (ev)	E_HOMO_ (ev)	V_oc_ (eV)/PC_60_BM	L_D_ − L_A(PC60BM_)	V_oc_ (eV)/PC_71_BM	L_D_ − L_A(PC71BM_)
P1	−3.057	−5.025	1.499	0.169	0.425	1.243
P2	−3.293	−5.276	1.75	−0.067	0.676	1.007
P3	−3.099	−5.091	1.565	0.127	0.491	1.201
P4	−3.124	−5.139	1.613	0.102	0.539	1.176
P5	−3.140	−5.155	1.629	0.086	0.555	1.160
P6	−3.159	−5.330	1.804	0.107	0.730	1.141
PC_61_BM	−3.226	−5.985	****	****	****	****
PC_71_BM	−4.300	−6.000	****	****	****	****

**Fig. 4:**
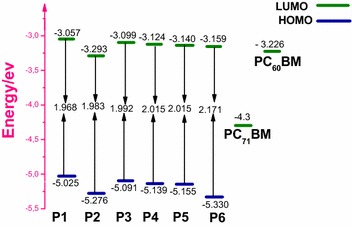
Sketch of B3LYP/6-31G(d,p) calculated energies of the HOMO, LUMO level of study molecules

In other side the Table 3 and the Fig. 4 show that the differences (L_D_ − L_A_) of LUMO energy levels between those new designed donors (P1, P2, P3, P4, P5 and P6) and the acceptor of PC_60_BM is larger than 0 eV except P2. The same remark in case PC_71_BM, the differences (L_D_ − L_A_) energy is also larger than 0 eV, which ensures efficient electron transfer from the donor to the acceptor (PC_60_BM, PC_71_BM) except P2 in case PC_60_BM because is more lower to 0 eV. This makes the transfer of electron from this compound (P2) to LUMO of PC_60_BM very difficult (LUMO of P2 is located below to LUMO of PC_60_BM).

Therefore, all the studied molecules can be used as BHJ because the electron injection process from the excited molecule to the conduction band of PCBM and the subsequent regeneration is possible in an organic sensitized solar cell.

**Fig. 5:**
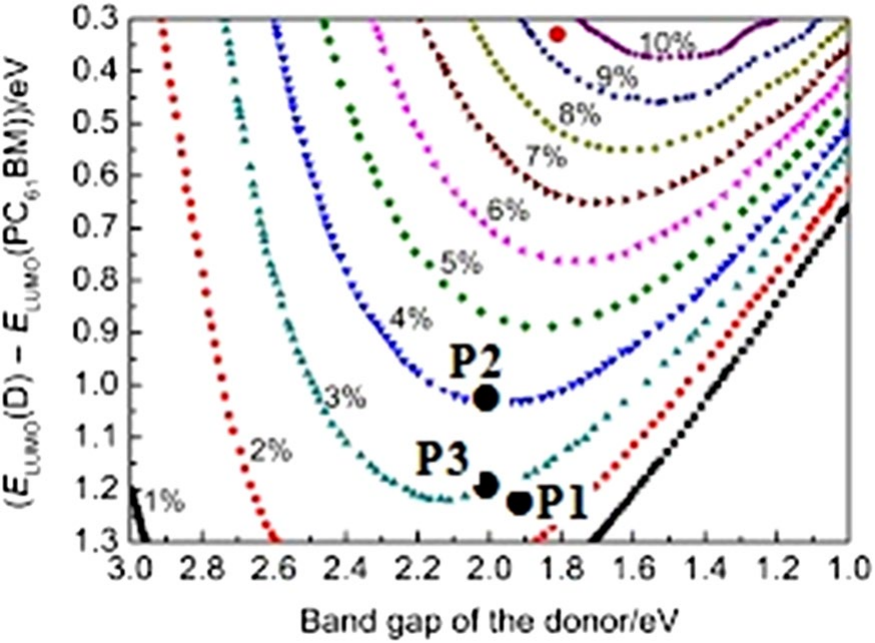
Calculated efficiency under AM1.5G illumination for single junction devices based on composites that consist of a donor with a variable band gap and LUMO level and an acceptor with a variable LUMO level [34]

It is possible to assess the ideal performance donor, according to the position of its [E_LUMO_ (donor) − E_LUMO_ (acceptor)] energy and its band gap (Fig. 5). Theoretically, a maximum energy conversion efficiency of about 10% could be achieved for CPOs [51, 52] an oligomer having a LUMO energy level between −3.8 and −4.0 eV and a band gap between 1.2 and 1.9 eV has a theoretical power conversion efficiency between 8 and 10%. In a tandem configuration, the combination of two polymers band gap of 1.8 eV and 1.5 or 1.5 and 1.2 eV in two active layers separated to increase the effectiveness of a complete device for achieving a conversion efficiency of energy theoretical about 15%. We note that the higher power conversion efficiency could be achieved for P_2_ is 4 and 3% for P_3_.

### Optical properties

**Fig. 6:**
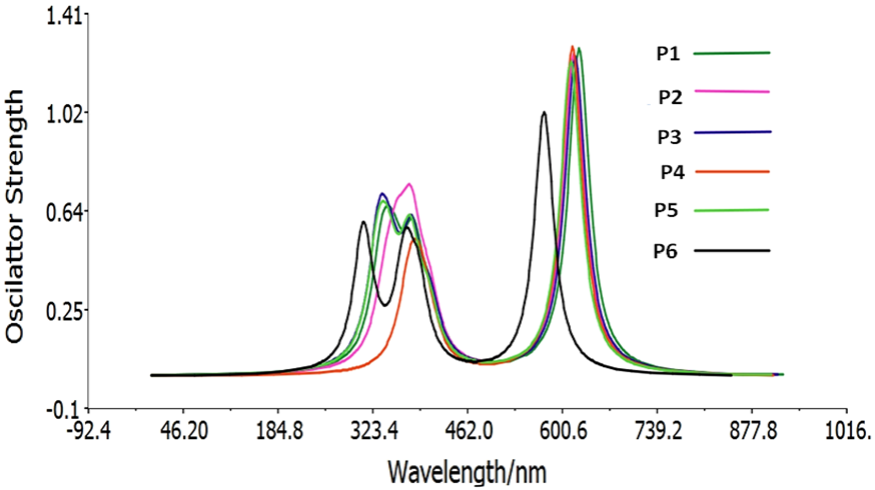
Simulated UV–visible optical absorption spectra of the title compounds with the calculated data at the TD-DFT/CAM-B3LYP/6-31G(d,p) level in chloroform solvent

To understand the electronic transitions from our compounds, the quantum calculation on electronic absorption spectra in the gaseous phase and solvent (chloroform) was performed using TD-DFT/CAM-B3LYP/6–31G(d, p) level. The calculated absorption wavelengths (*ʎ*_max_), oscillator strengths (ƒ) and vertical excitation energies (E) for gaseous phase and solvent (chloroform) were carried out and listed in Table 4. The spectra show a similar profile for all compounds which present a main intense band at higher energies from 548.16 to 591.46 nm for gas phase and 574.33 to 625.38 for chloroform solution and were assigned to the ICT transitions. From Table 4, we could find that as the donor group changing, the first vertical excitation energies (E) were changed in decreasing order in both phases (gaseous and solvated): P6 > D5 > P4 > P2 > P3 > P1 showing that there is a red shift when passing from P6 to P1. We remark that the transition which has the larger oscillator strength is the most probable transition from the ground state to an excited state of all transitions, corresponding to excitation from HOMO to LUMO of gas phase and chloroform solution, This electronic absorption corresponds to the transition from the molecular orbital HOMO to the LUMO excited state, is a π–π* transition. These results indicate that all molecules have only one band in the Visible region (λ_abs_ > 400 nm) (Fig. 6) and P1 could harvest more light at the longer-wavelength which is beneficial to further increase the photo-to-electric conversion efficiency of the corresponding solar cells. So the lowest lying transition can be tuned by the different π-spacer.

**Table 4:** Absorption spectra data obtained by TD-DFT methods for the title compounds at CAM-B3LYP/6-31G(d,p) optimized geometries in the gas phase and in solvent phase (chloroform)

Compounds	In the gas phase			In solvent phase			MO/character
	λ_abs_ (nm)	E_ex_ (eV)	ƒ	λ_abs_ (nm)	E_ex_ (eV)	ƒ	
P1	591.46	2.0963	1.0923	625.38	1.9826	1.2732	HOMO → LUMO
P2	584.40	2.1215	1.0513	618.01	2.0062	1.2540	HOMO → LUMO
P3	585.30	2.1183	1.0564	620.04	1.9996	1.2416	HOMO → LUMO
P4	581.15	2.1334	1.1148	615.49	2.0144	1.2817	HOMO → LUMO
P5	580.40	2.1362	1.0411	613.46	2.0211	1.2234	HOMO → LUMO
P6	548.16	2.2618	0.8707	574.33	2.1587	1.0239	HOMO → LUMO

In order to study the emission photoluminescence properties of the studied compounds Pi (i = 1 to 6), the TDDFT/CAM-B3LYP method was applied to the geometry of the lowest singlet excited state optimized at the CAM-B3LYP/6–31 (d, p), and the theoretical emission calculations with the strongest oscillator are presented in Table 5. The emission spectra arising from the S1 state is assigned to π* → π and LUMO → HOMO transition character for all molecules. Through analyzing the transition configuration of the fluorescence, we found that the calculated fluorescence has been just the reverse processed of the lowest lying absorption. Moreover, the observed red-shifted emission of the photoluminescence (PL) spectra when passing from P1 to P6 is in reasonable agreement with the obtained results of absorption. We can also note that relatively high values of Stocks Shift (SS) are obtained from all compounds P1 (179.64 nm), P2 (176.64), P3 (181.49 nm), P4 (178.33 nm), P5 (177.26 nm) and P6 (152.68 nm) (Table 5), this indicate that the compounds which have a weak Stocks Shift present a minimal conformational reorganization between ground state and excited state. Indeed, this stops the intermolecular transfer charge and delaying the injection phenomenon from LUMO of the compounds to LUMO of PCBM. In fact, the Stokes shift, which is defined as the difference between the absorption and emission maximums (E_VA_–E_VE_), is usually related to the bandwidths of both absorption and emission bands [53].

**Table 5:** Emission spectra data obtained by TD-DFT methods for the title compounds at B3LYP/6–31G(d,p) optimized geometries in chloroform solvent

Compounds	Excited state	Main composition	MO	ʎ_max_ emis (nm)	ΔE (eV)	ƒ	Radiative life times (ns)	SS
P1	S1 S0	LUMO → HOMO	0.69404	805.02	1.5401	1.3298	7.33	179.64
P2	S1 S0	LUMO → HOMO	0.68889	794.65	1.5602	1.2922	7.35	176.64
P3	S1 S0	LUMO → HOMO	0.69578	801.53	1.5468	1.3050	7.40	181.49
P4	S1 S0	LUMO → HOMO	0.68760	793.82	1.5619	1.3328	7.11	178.33
P5	S1 S0	LUMO → HOMO	0.69658	790.72	1.5680	1.2771	7.36	177.26
P6	S1 S0	LUMO → HOMO	0.69912	727.01	1.7054	1.0439	7.61	152.68

### Excited state lifetimes

The radiative lifetimes (in au) have been computed for spontaneous emission using the Einstein transition probabilities according to the following formula [54]:

3 $$\tau ={C^{3} } {\bigg/}{2(E_{Flu} )^{2} {\text{f}}}$$

where (c) is the velocity of light, E_*Flu*_ is the excitation energy, and ƒ is the oscillator strength (O.S.). The computed lifetimes (τ), for the title compounds are listed in Table 5. However, an increase in lifetimes of Pi will retard the charge recombination process and enhance the efficiency of the photovoltaics cells. So, long radiative lifetimes facilitate the electron transfer upon the photoexcited electron, from LUMO of electron-donor to LUMO of electron-acceptor, thus lead to high light-emitting efficiency. The radiative lifetimes of the study compounds are from 7.61 to 7.11 ns and increases in the following order P4 < P1 < P2 < P5 < P3 < P6. This result is sufficient to obtain a high light-emitting efficiency, especially for P6.

## Conclusions

We have used the density functional theory method to investigate the geometries and electronic properties of some thienopyrazine-derivatives in alternate donor-π-acceptor structure. The modification of chemical structures can greatly modulate and improve the electronic and optical properties of pristine studied materials. The electronic properties of new conjugated materials based on thienopyrazine and heterocyclic compounds and different acceptor moieties have been computed by using 6-31G(d,p) basis set at a density functional B3LYP level, in order to guide the synthesis of novel materials with specific electronic properties. The concluding remarks are:

The predicted band gaps by using DFT-B3LYP/6-31G(d,p) are in the range of 1.968–2.171 eV, knowing that the small band gap due to the increasing of the displacement of the electron between donor and acceptor spacer is very easy. The much lower Eg of P1, P2, and P3 compared to other compounds a significant effect of intramolecular charge transfer. However, the Eg values of P1, P2 and P3 are smaller than that of P6.

The theoretical values of the open circuit voltage V_oc_ of the studied molecules range from 1.499 to 1.804 eV in the case of PC_60_BM and 0.425 to 0.73 eV in the case of PC_71_BM, these values are sufficient for a possible efficient electron injection. After the results, we note that all the studied molecules can be used as BHJ because the electron injection process from the excited molecule to the conduction band of PCBM and the subsequent regeneration is possible in an organic sensitized solar cell. It is concluded that We note that the higher power conversion efficiency could be achieved for P_2_ is 4 and 3% for P_3_.

The TD-DFT calculations, at least TD-CAM-B3LYP/6-31G(d,p) was used to replicate the optical transitions in order to predict the excited and emission states; the predicted result of the absorption wavelengths for P1, P2, P3, P4, P5, and P6 is 805.02, 794.65, 801.53, 793.82, 790.72 and 727.01 nm respectively.

The decreasing of the band gap of these six materials due to increasing the absorption wavelengths, then the best commands which can be used in photovoltaic cells such as donor of electronic, is one which has the small band gap and large wavelengths, thus all compounds (1–6) are appropriate to do this role.
